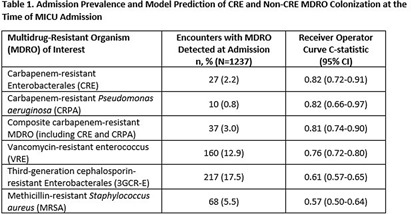# Application of a Model Using Prior Healthcare Information to Predict Multidrug-Resistant Organism (MDRO) Carriage

**DOI:** 10.1017/ash.2024.266

**Published:** 2024-09-16

**Authors:** Sarah Sansom, Michael Lin, Anh-Thu Runez, Dejan Jovanov, Helen Zhang, Michael Schoeny, Mary Hayden, William Trick

**Affiliations:** Rush University Medical Center; Illinois Department of Public Health; Cook County Health

## Abstract

**Background:** Early identification of patients colonized with MDROs can help healthcare facilities improve infection control and treatment. We evaluated whether a model previously validated to predict carbapenem-resistant Enterobacterales (CRE) carriage on hospital admission (area under the curve [AUC]=0.86, Lin et al. OFID 2019) would generalize to predict a patient’s likelihood of CRE and non-CRE MDRO colonization at the time of medical intensive care unit (MICU) admission. **Methods:** We analyzed data collected previously in a retrospective observational cohort study of patients admitted to Rush University Medical Center’s MICU from 1/2017-1/2018 and screened within the first two days for rectal MDRO colonization. Organisms of interest included CRE, carbapenem-resistant Pseudomonas aeruginosa (CRPA), vancomycin-resistant enterococci (VRE), and third-generation cephalosporin-resistant Enterobacterales (3GCR-E). Methicillin-resistant Staphylococcus aureus (MRSA) nasal colonization at admission was determined by routine clinical screening. Each patient’s first MICU admission during the study period was linked to Illinois’ hospital discharge database and assigned a CRE colonization risk probability using the existing model. Model covariates were age, and during the prior 365 days, number of short-term acute care hospitalizations (STACH) and mean STACH length of stay, number of long-term acute care hospitalizations (LTACH) and mean LTACH length of stay, prior hospital admission with an ICD-10 diagnosis code indicating bacterial infection, and current admission to LTACH. Predictive value of the model was evaluated by receiver operating characteristic (ROC) curves. **Results:** We analyzed 1237 MICU admissions. MDRO admission prevalence is shown in the Table. The model performed well to predict carriage of healthcare-associated MDROs, including CRE, CRPA, composite CR-MDROs (CRE & CRPA), and VRE. However, the model performed poorly for MDROs with known community reservoirs, including 3GCR-E and MRSA (Table). In general, MDRO admission prevalence increased in parallel with predicted CRE colonization risk (Figure). The number needed to screen (NNS) to detect one healthcare-associated MDRO carrier was inversely related to the CRE colonization risk score. For example, NNS in the total cohort compared to those with CRE risk score of >0.5% was: CRE 111 vs 32 patients, CRPA 333 vs 42 patients, composite CR-MDRO 83 vs 18 patients, and VRE 12 vs 4 patients. However, higher CRE risk score cutoff was inversely related to screening sensitivity. **Conclusion:** A prediction model using prior healthcare exposure information successfully discriminated patients likely to harbor healthcare-associated MDROs upon MICU admission. Prediction scores generated by a public-health accessible database could be used to target screening/isolation or enact protective measures for high-risk patients.

**Figure:**
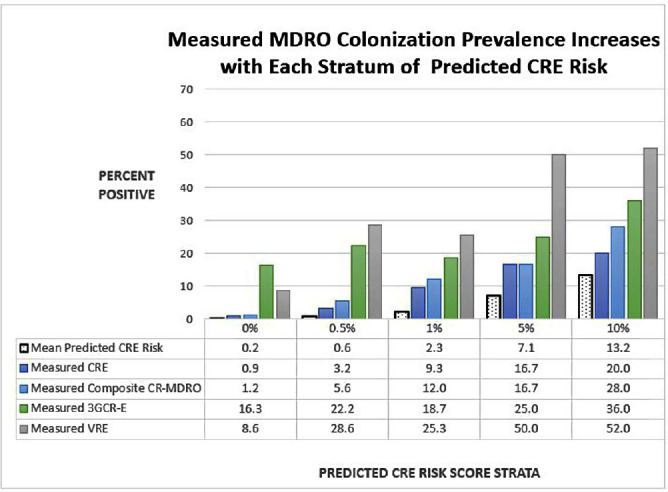

**Figure:**